# Germinating fission yeast spores delay in G1 in response to UV irradiation

**DOI:** 10.1186/1471-2121-5-40

**Published:** 2004-10-21

**Authors:** Esben A Nilssen, Marianne Synnes, Tonje Tvegård, Heidi Vebø, Erik Boye, Beáta Grallert

**Affiliations:** 1 Department of Cell Biology, Institute for Cancer Research, Montebello, 0310 Oslo, Norway

## Abstract

**Background:**

Checkpoint mechanisms prevent cell cycle transitions until previous events have been completed or damaged DNA has been repaired. In fission yeast, checkpoint mechanisms are known to regulate entry into mitosis, but so far no checkpoint inhibiting S phase entry has been identified.

**Results:**

We have studied the response of germinating *Schizosaccharomyces pombe *spores to UV irradiation in G1. When germinating spores are irradiated in early G1 phase, entry into S phase is delayed. We argue that the observed delay is caused by two separate mechanisms. The first takes place before entry into S phase, does not depend on the checkpoint proteins Rad3, Cds1 and Chk1 and is independent of Cdc2 phosphorylation. Furthermore, it is not dependent upon inhibiting the Cdc10-dependent transcription required for S phase entry, unlike a G1/S checkpoint described in budding yeast. We show that expression of Cdt1, a protein essential for initiation of DNA replication, is delayed upon UV irradiation. The second part of the delay occurs after entry into S phase and depends on Rad3 and Cds1 and is probably due to the intra-S checkpoint. If the germinating spores are irradiated in late G1, they enter S phase without delay and arrest in S phase, suggesting that the delay we observe upon UV irradiation in early G1 is not caused by nonspecific effects of UV irradiation.

**Conclusions:**

We have studied the response of germinating *S. pombe *spores to UV irradiation in G1 and shown that S phase entry is delayed by a mechanism that is different from classical checkpoint responses. Our results point to a mechanism delaying expression of proteins required for S phase entry.

## Background

Checkpoint mechanisms are important for cell survival and genetic stability. They prevent cell cycle transitions until previous events have been completed or damaged DNA has been repaired [1]. Checkpoint pathways and proteins are evolutionarily conserved from yeast to man, underlining their importance in maintaining genomic integrity. In fission yeast several checkpoint pathways monitor the status of the DNA and arrest the cell cycle in response to DNA damage or inhibition of DNA replication [2,3] They include mechanisms to inhibit mitosis when the DNA is damaged (the G2/M checkpoint) or when S phase has not been completed (the S/M checkpoint) as well as a mechanism to inhibit ongoing DNA replication when the DNA is damaged (the intra-S checkpoint). Screens designed to reveal elements of the checkpoint pathways have led to the identification of the so-called checkpoint *rad *genes as well as *crb2*/*rhp9*, *mrc1*, *chk1 *and *cds1 *[4-13] The checkpoint *rad *genes consist of *rad1*, *rad3*, *rad9*, *rad17*, *rad26 *and *hus1 *(reviewed in ([14]). Rad3 is a member of the phosphatidylinositol 3-kinase family of proteins and the closest mammalian homologue is the ATR (ATM and Rad3 related) protein [15,16]. Rad3 forms a complex with Rad26 and this association is required for activation of Rad3 kinase activity in response to DNA damage or replication arrest [17,18] The Rad1, Rad9 and Hus1 proteins have similarities to PCNA, the sliding clamp of the replicative DNA polymerase, and the three proteins may form a similar ring-shaped structure [19-21]. Rad17 has similarities to all five subunits of replication factor C [22], a complex which loads PCNA onto chromatin.

There are two known effector kinases downstream of the checkpoint Rad proteins, Chk1 and Cds1. Chk1 is phosphorylated in response to DNA damage induced in late S or G_2 _in a Rad3 dependent manner [12,23,24]. Phosphorylation of Chk1 leads to an increase of Chk1 kinase activity [25] and is often used as a convenient molecular marker for Chk1 dependent checkpoint activation. Cds1 is activated only in S phase as part of the intra-S and the S/M checkpoints [8,26] Activation of either kinase leads to inhibition of Cdc2 activity by maintaining the inhibitory phosphorylation on Tyr15 [27-29].

Crb2 and Mrc1 act upstream of Chk1 and Cds1, respectively. Crb2 shares homology with the budding yeast RAD9 protein [10], which is involved in delaying entry into S phase upon DNA damage in G1 [30-32] In fission yeast, Crb2 is required both for activation of Chk1 and for subsequent inactivation of Chk1 for reentry into the cell cycle [10,33] Mrc1 plays a parallel role by binding to and activating Cds1. Expression of Mrc1 is regulated in the cell cycle, thus linking Cds1 activation to S phase [6,11].

In addition to the G2/M, S/M and intra-S checkpoints, three papers have reported the existence of G1 checkpoints that inhibit mitosis when the cells are arrested in G1 using cell cycle mutants. Arrest at the *cdc10 *arrest point was shown to depend on Chk1 [34] and Rum1 [35]. Arrest of *orp1 *mutant cells depends on the checkpoint Rad proteins and on Chk1 [36]. It should be noted that neither of these cell cycle mutants is able to replicate their DNA at the restrictive temperature, and failure of the checkpoints responsible for cell cycle arrest results in aberrant entry into mitosis and not into S phase.

A G1/S checkpoint has so far not been detected in *S. pombe*. The drop of CDK activity at the M/G1 transition allows the assembly of the pre-Replication Complex, preRC, which is the first step leading to initiation of S phase. The preRC consists of the ORC (Origin Recognition Complex), Cdc18, Cdt1 and the MCM proteins. Expression of Cdc18 and Cdt1 is cell cycle regulated, thus providing one of the means to regulate initiation of S phase [37,38] Once the preRC is assembled, the chromatin is competent to replicate, but replication is not initiated until other replication proteins are loaded, and two kinases, Cdc2 and Hsk1, are activated. It has been shown both in fission yeast and in *Xenopus *that the intra-S phase checkpoint cannot be engaged until polα-primase is loaded and replication begins [39,40] This observation poses the question whether the cells have any means to respond to DNA damage sustained in G1.

G1 in fission yeast is very short under standard laboratory growth conditions, rendering the investigation of a G1/S checkpoint(s) difficult. However, G1 might be much extended in the natural habitat of *S. pombe *due to poor nutrient availability. We decided to use several approaches to synchronise the cells and/or to extend G1. Recently we reported the existence of a mechanism that delays entry into S phase when cycling cells are UV-irradiated in G1, using *cdc10 *and *cdc25 *mutants to synchronise the cells or growing the cells in medium where G1 is extended [41].

Here we show that germinating *S. pombe *spores delay entry into S phase upon UV irradiation in early but not late G1. We demonstrate that there is a G1/S delay that is not dependent on any of the known checkpoint proteins and does not target Cdc2 phosphorylation. We argue that the delay is due to a novel mechanism that leads to delayed expression of Cdt1 and possibly other replication proteins.

## Results

### Entry into S phase is delayed by UV irradiation

Spores made from diploid cells were allowed to germinate for 3.5 h at 30°C before UV irradiation. At this time point, 1 – 2 hours before S phase entry, the spores showed visible signs of germination by phase contrast microscopy. The dose of UV light was 1200 J/m^2^, which gave a cell survival of about 30% in wild type cells (data not shown). At the time of irradiation, the majority of germinating spores had a 1C DNA content. The timing of S phase was measured, by flow cytometry, as an increase in cellular DNA content from 1C to 2C. The decrease of the 1C population was plotted against time, and the graphs for unirradiated control and UV irradiated cells were compared at the point where 50% of the cells had 1C DNA content.

**Figure 1:**
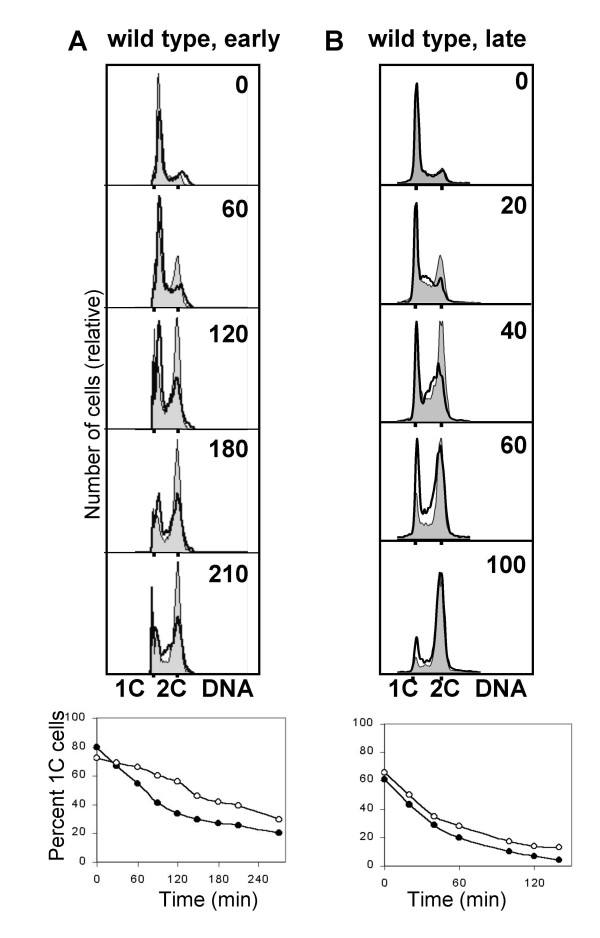
Irradiation of germinating wild type spores delays entry into S phase. Germinating spores were irradiated with UV light 3.5 h (**A**) and 4.5 h (**B**) (time 0) after inoculation into EMM medium, as described in Materials and Methods. Samples were taken for flow cytometry at the indicated times after treatment. The uppper panels show DNA histograms for the unirradiated control (shaded) and the irradiated cells (bold outline without shading). The lower panels show the quantification of cells with a 1C DNA content. Filled symbols represent the control cells, open symbols represent the irradiated cells.

An inherent problem in the present experiments is that the time of germination varies both within each population of spores (low degree of synchrony) and between the different preparations (experiment-to-experiment variation). Thus, the time from resuspension in medium until the cells enter S phase is variable, and it is difficult to ensure that irradiation occurs at exactly the same time point relative to S phase entry. Therefore the experiments were repeated at least twice and the averages were determined (Table 1). The experiments revealed that UV irradiation made wild type cells delay their S phase entry by 76 minutes relative to unirradiated control cells (Fig. 1A, Table 1). When the irradiated cells started to increase their DNA content, they did not delay appreciably within S phase compared to unirradiated control cells, suggesting that when they started to synthesise DNA, the DNA damage had been removed. However, when the germinating spores were irradiated shortly before S phase entry, they entered S phase without a delay and were arrested with a DNA content between 1C and 2C (Fig. 1B, Table 1), presumably due to the intra-S checkpoint. Indeed, the later the irradiation was performed the more pronounced the intra-S phase delay was (data not shown). We conclude that cells irradiated in early G1 arrest temporarily with 1C DNA content, then replicate their DNA with normal timing. Cells irradiated in late G1 exit from 1C with the same kinetics as unirradiated control cells do, but they are unable to complete S phase in normal time.

**Table 1:** Length of the delay in the investigated mutants

**Mutant**	**Length of the 1C delay (min)**(*)	**Average length of the 1C delay (min)**
wt early irradiation	70, 80, 90, 60, 80	76
wt late irradiation	<10, <10	<10
caffeine	<10, <10	<10
*rad3*	40, 20, 45, 15	30
*rad26*	45, 40	43
*rad1*	50, 40	45
*rad9*	40, 30	35
*hus1*	55, 40	50
*rad17*	40, 30	35
*cds1*	50, 40	45
*chk1*	90, 55, 70	72
*chk1 cds1*	35, 40	38
*rum1*	55, 45, 25	42
*res2*	<10, <10, <10	<10

(*) The length of the delay was measured at the point where 50% of the cells had a 1C DNA content on the quantitations. The second column shows the results of individual experiments, the third column shows the average lengths of the delay. Irradiations were carried out 3.5 hours after inoculation in medium, except for the entry "wt late irradiation", which was performed 4.5 hours after inoculation.

### The cells delay with low levels of Cdt1

**Figure 2:**
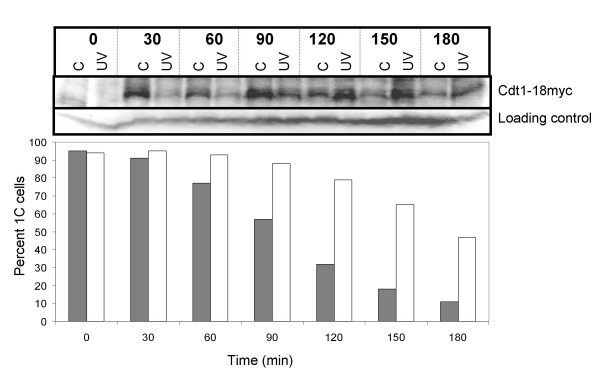
The cells delay with low levels of Cdt1. Wild type spores carrying myc tagged Cdt1 were germinated and irradiated as described in the legend to Figure 1A. Samples were taken for protein extracts and flow cytometry at the times indicated. Total protein extracts were prepared and the amount of Cdt1-myc was investigated by SDS-PAGE and immunoblot analyses against total Cdc2, which served as loading control, and Cdt1-myc (top panel). Quantification of the fraction of cells with a 1C DNA content is also shown in the bottom panel (filled symbols: control; open symbols: UV).

Flow cytometry cannot distinguish between G1 and early S phase cells, therefore we sought to confirm that the cells arrest prior to S phase. PreRC formation is a prerequisite for initiation of S phase. The first step towards preRC formation is *de novo *synthesis of Cdc18 and Cdt1, which in turn are required for MCM loading. We investigated Cdt1 levels in germinated spores treated as above to establish the timing of the 1C delay relative to Cdt1 expression. Wild type spores carrying myc-tagged Cdt1 were UV irradiated as described above and samples of irradiated and control cells were removed for analysis by flow cytometry and by immunoblotting. Cdt1 expression was induced already at 30 minutes in the control cells, but not until 55 minutes later in the UV irradiated cells (Fig. 2, Table 2). These observations suggest that cells irradiated in early G1 may delay entry into S phase at least in part by delaying preRC formation.

**Table 2:** Cdt1 expression and Cdc2 phosphorylation are delayed upon UV irradiation

**Event**	**Length of the delay (min)**(*)	**Average length of the delay (min)**
Cdt1 expression	60, 50	55
Cdc2 phosphorylation	40, 50	45

(*) The levels of Cdt1 expression and Cdc2 phosphorylation were quantified and the length of the delay was measured at the point where Cdt1 expression and Cdc2 phosphorylation, respectively, reached 50% of its maximal value. Irradiations were carried out 3.5 hours after inoculation in medium.

### Rum1 is required for part of the delay

**Figure 3:**
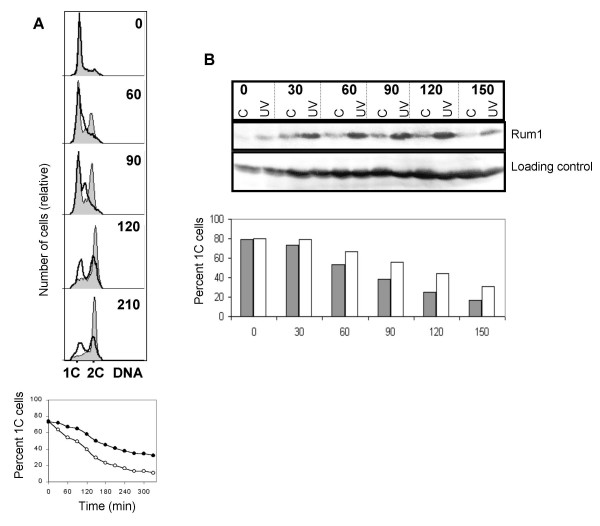
Rum1 is required for part of the delay. **A. ***rum1Δ *spores were irradiated and analysed as described in the legend to Figure 1A. **B**. Wild type spores were germinated and irradiated as described in the legend to Figure 1A. Samples were taken for protein extracts and flow cytometry at the times indicated. Total protein extracts were prepared and the amount of Rum1 was investigated by SDS-PAGE and immunoblot analyses against total Cdc2, which served as loading control, and Rum1. Quantification of the fraction of cells with a 1C DNA content is also shown in the bottom panel (filled symbols: control; open symbols: UV).

Rum1 inhibits the mitotic CDK, Cdc2-Cdc13, and is required for efficient proteolysis of Cdc13 [42-44]. Furthermore, Rum1 is required for all G1 arrests and delays investigated so far. We irradiated germinating *rum1Δ *spores as above and progression into S phase was followed by flow cytometry. Irradiated spores delayed with a 1C DNA content for 40 minutes (Fig. 3A, Table 1). At the 60–90 minute timepoints the irradiated cells display a distinct delay in S phase, consistent with activation of the intra-S-phase checkpoint. The absence of Rum1 shortens G1, therefore some of the germinating spores were in fact in late G1 or early S at the time of irradiation, giving rise to significant activation of the intra-S-phase checkpoint.

Rum1 expression is cell cycle regulated such that it is only expressed in G1 [45]. We investigated Rum1 levels in germinated spores UV irradiated as described above and samples of irradiated and control cells were removed for analysis by flow cytometry and by immunoblotting. Rum1 expression was induced at 30 minutes in both cultures, but was maintained to a higher extent and longer in the irradiated cells (Fig. 3B). Both increased expression of Rum1 and the requirement for Rum1 for part of the delay demonstrate that part of the delay takes place in G1.

### Is the 1C delay checkpoint dependent?

**Figure 4:**
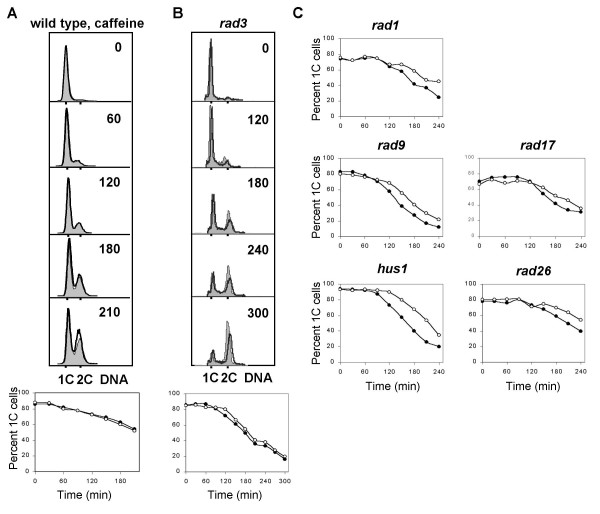
Checkpoint Rad proteins in the G1 checkpoint. **A. **Wild type spores germinating in the presence of caffeine were irradiated and analysed as described in the legend to Figure 1A. **B. **Germinating *rad3 *spores were irradiated and analysed as described in the legend to Figure 1A. **C**. The indicated mutants were sporulated and the germinating spores were irradiated and analysed as described in the legend to Figure 1A.

The definition of a checkpoint calls for the existence of mutations or chemicals that eliminate the delay. We addressed this issue by treating the germinating spores with caffeine. Caffeine is known to abolish checkpoint function in both higher eukaryotes and fission yeast, possibly through the inhibition of Rad3 [46]. Caffeine was added to the culture 15 minutes before UV irradiation. Flow cytometric analyses showed that the caffeine-treated spores entered S phase with the same kinetics as unirradiated cells (Fig. 4A, Table 1). This observation indicates, (but does not prove, see Discussion), that the delay might be caused by a checkpoint mechanism.

Given that caffeine can inhibit Rad3 related kinases, we investigated whether the G1/S delay is also abolished in *rad3 *mutant cells. Irradiated *rad3 *germinating spores delayed with a 1C DNA content for 30 minutes, in contrast to the 76 minute delay of wild type cells (Fig. 4B, Table 1).

We have investigated whether the other checkpoint Rad proteins are involved in the G1/S delay. *rad26*, *rad1*, *rad9*, *hus1*, and *rad17 *spores were germinated and UV irradiated as described above. Figure 4C (and Table 1) shows that *rad26*, *rad1*, *rad9*, *hus1 *and *rad17 *cells delay much less than wild type cells do (35–50 versus 76 min). We conclude that Rad26, Rad1, Rad9, Hus1 and Rad17 are required for at least a part of the 1C delay.

### Mrc1 and Cds1, but not Crb2 and Chk1, are required for part of the delay

**Figure 5:**
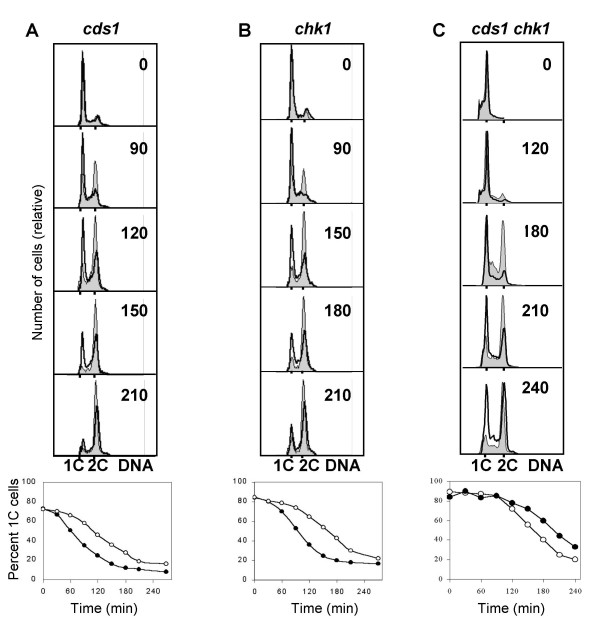
Cds1, but not Chk1, is required for part of the delay. *cds1Δ *(**A**), *chk1Δ *(**B**) and *chk1Δ cds1Δ *(**C**) spores were irradiated and analysed as described in the legend to Figure 1A.

The products of the checkpoint genes *cds1 *and *chk1 *are both known downstream targets of the Rad3 protein kinase and they are required for Cdc2 phosphorylation in the DNA damage and replication checkpoints. We irradiated germinating spores carrying mutations of *cds1*, *chk1 *or both. In *cds1 *spores the delay was reduced to 45 minutes (Fig. 5A, Table 1). In *chk1 *spores (Fig. 5B, Table 1) the length of the delay was not reduced compared to that found in wild type cells. In *cds1 chk1 *double mutant spores the delay was somewhat shorter than in either single mutant, 35 minutes *versus *45 and 73 minutes (Fig. 5C, Table 1). The shorter delay in the *cds1 chk1 *double mutant compared to that in *cds1 *indicates that *chk1 *might have a synthetic effect with the *cds1 *mutation.

Crb2 and Mrc1 are required for activation of Chk1 and Cds1, respectively. We irradiated germinating *crb2 *and *mrc1 *spores [11]. Consistent with the above findings, in *mrc1 *the delay was reduced to 50 minutes, while in *crb2 *the delay was not reduced compared to that in wild type cells (data not shown). We conclude that Mrc1 and Cds1 are required for part of the delay, while Crb2 and Chk1 are not required.

### The arrested cells maintain Cdc2 in the unphosphorylated form

**Figure 6:**
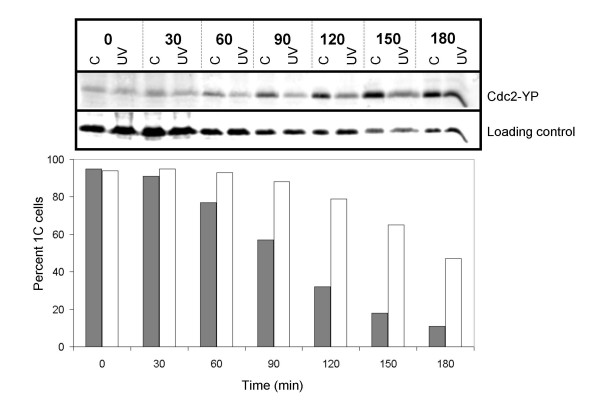
Cdc2 is not phosphorylated in the arrested cells. Wild type spores were germinated and irradiated as described in the legend to Figure 1A. Samples were taken for protein extracts and flow cytometry at the times indicated. Total protein extracts were prepared and the amount of phosphorylated Cdc2 was investigated by SDS-PAGE and immunoblot analyses against total Cdc2, which served as loading control, and phosphorylated Cdc2 (top panel). Quantification of the fraction of cells with a 1C DNA content is also shown in the bottom panel (filled symbols: control; open symbols: UV).

Cdc2 kinase activity is required for the initiation of S phase and is inhibited by phosphorylation on Tyr15 as DNA replication commences [35,47]. We investigated whether the Cdc2 protein is phosphorylated when the cells are delayed with a 1C DNA content. Germinating wild type spores were treated as above and samples of irradiated and control cells were removed for analysis by flow cytometry and by immunoblotting. The results show an increase in the phosphorylation signal as the unirradiated cells enter S phase (Fig. 6), in agreement with previous findings [35,47] The irradiated cells increased phosphorylation of Cdc2 45 minutes later (Table 2). We conclude that the irradiated cells arrest with a 1C DNA content for a significant length of time with unphosphorylated Cdc2.

### *res2 *mutant cells do not delay S phase entry after irradiation

**Figure 7:**
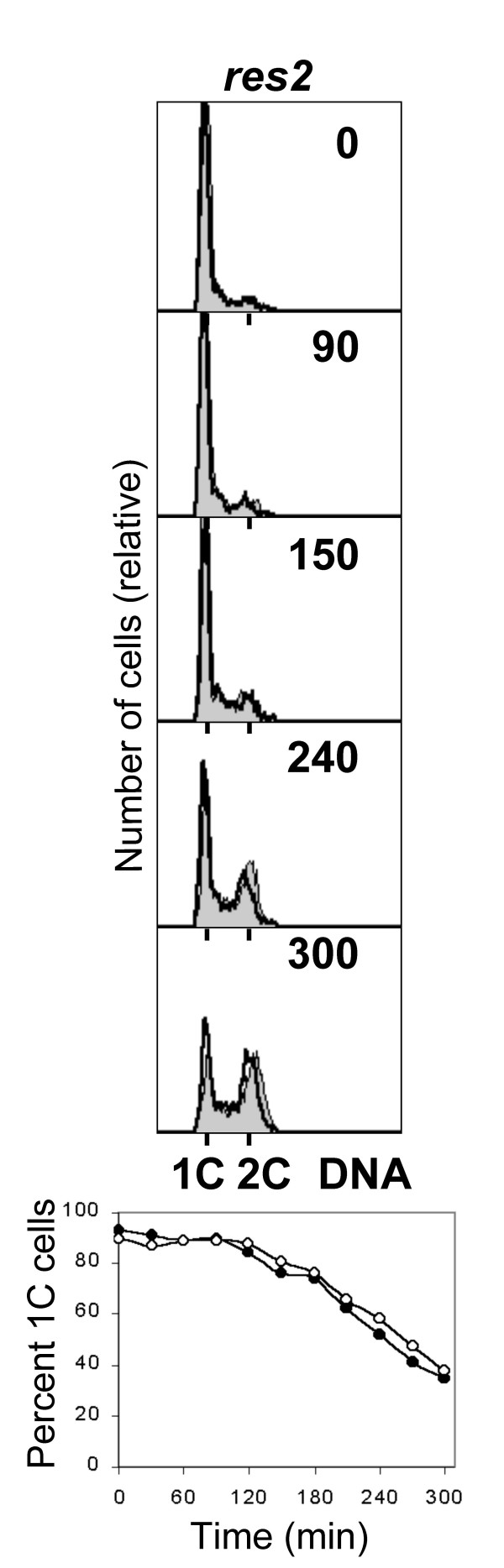
The *res2Δ *mutant cells do not display the delay. *res2Δ *mutant spores were germinated and irradiated as described in the legend to Figure 1A.

A number of genes required for DNA replication are transcribed as the cells prepare for S phase. This activation depends on the cell cycle regulated transcription factor Cdc10/Res1/Res2 [48,49]. In the absence of Res2, transcription is constitutively active [50]. If the G1/S delay in fission yeast cells is brought about by inhibiting this transcription factor, constitutive activation of transcription in a *res2 *mutant should override the UV-induced G1 delay. We have irradiated germinating *res2Δ *spores as described above and found that S phase entry was not delayed compared to unirradiated control cells (Fig. 7, Table 1).

### Cdc10 dependent transcription is not inhibited during the delay

**Figure 8:**
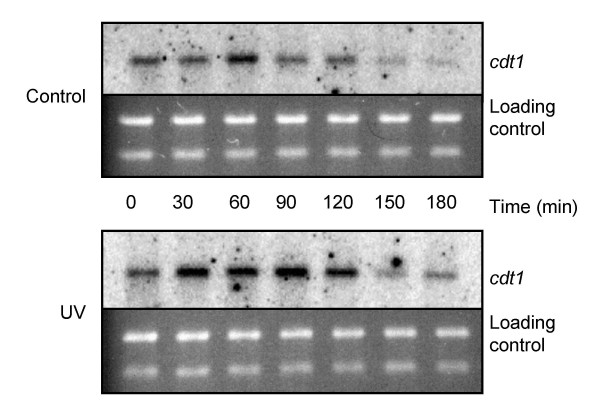
Cdc10 dependent transcription is not inhibited during the delay. Wild type spores were germinated and irradiated as described in the legend to Figure 1A. Samples were taken for RNA extracts and flow cytometry at the indicated times. Total RNA extracts were prepared and the amount of *cdt1 *mRNA was investigated by Northern analysis.

The above result indicates that either constitutive expression of Cdc10 dependent genes required for S phase entry can override the delay or inhibiting Cdc10 dependent transcription might be the mechanism of the delay. A prediction of the latter alternative is that cells arrested with 1C DNA content upon UV irradiation should not have performed the Cdc10-dependent transcriptional events, including induction of the *cdc18*, *cdt1*, and *cig2 *genes. We isolated total RNA from irradiated and unirradiated germinating wild type spores and followed the transcription of *cig2*, *cdt1 *and *cdc18*. There was no delay in the appearance of the Cdc10 dependent transcripts upon UV irradiation (Fig. 8, only data for *cdt1 *are shown). We conclude that Cdc10 dependent transcription is not the mechanism of the delay.

## Discussion

We have provided evidence for the existence of a mechanism in germinating fission yeast spores that delays entry into S phase upon UV irradiaton in early G1. Germinating wild type spores displayed a pronounced delay in entering S phase after UV irradiation. The delay was observed only when irradiation was carried out in early but not in late G1. We have investigated the dependence of the delay on classical checkpoint proteins and showed that they are required for some but not all of the delay with 1C DNA content. We argue that the observed delay is caused by two separate mechanisms, the first taking place before entry into S phase, and the second in early S phase (see below).

The delay in exit from the 1C population was demonstrated by means of flow cytometry, which does not allow us to distinguish between a G1/S and an early S delay. The following data represent strong evidence that part of the delay takes place before entry into S phase. First, the irradiated cells delay expression of Cdt1. In the absence of Cdt1 the cells cannot form preRCs and thus cannot initiate S phase. Second, the irradiated cells express Rum1 longer than unirradiated control cells. Since Rum1 expression is cell cycle regulated such that it is only expressed in G1, [45], this observation implies that the irradiated cells do delay in G1. Furthermore, the delay is shorter in a *rum1 *mutant, which presumably loses the G1 part of the delay. Third, mutants lacking Mrc1 or Cds1, which are essential for S-phase checkpoints reported so far in fission yeast [6], still delay for a significant length of time, pointing to the existence of a non-S mechanism [11]. Fourth, cells delay with the Cdc2 kinase in an unphosphorylated state. Cdc2 is normally inhibited by phosphorylation on Tyr15 as DNA replication commences [35,47], arguing that the cells arrest before S phase. Fifth, the delay is not observed in a *res2 *mutant, which can not turn off the Cdc10 dependent transcription signal. The finding that a mutation affecting expression of proteins crucial for preparation for S phase abolishes the delay argues that the wild type cells first stop in G1 and only later stop in S phase.

On the basis of these results we conclude that there is a UV-induced G1 delay, which is not checkpoint Rad dependent and is brought about by an as yet undescribed mechanism. This part of the total delay with 1C DNA content is ca 40 minutes, since *rad*, *cds1 *and *mrc1 *mutants delay 30–50 minutes and *rum1Δ *cells lose ca 40 minutes of the delay, compared to wild type cells. The remaining ca 40 minutes of the total delay requires the checkpoint Rads, Mrc1, Cds1 and Cdc2 is phosphorylated. We argue that this part of the delay is brought about by the intra-S checkpoint. However, the resolution of our experiments is not high enough to exclude the possibility that some of the checkpoint Rad- and Cds1-dependent part of the delay occurs in late G1. We consider this possibility unlikely for two reasons; first, previous work has shown that the role of Cds1 is specific for S phase [26] and second, we have shown in the current paper that if irradiation occurs later, the cells enter S phase without delay and delay in S phase.

Since the level of synchrony is low in germinating spores, we have not emphasised minor differences in the timing of S phase entry. In spite of poor synchrony, we deem germinating spores a good model system, since spore germination is a natural phenomenon, it involves an extended G1 period and we observed clear-cut effects. Furthermore, this model system allowed us to investigate the effects of a number of mutations that would have not been possible using synchronisation by other methods.

We have explored whether the G1/S delay is caused by a checkpoint mechanism. We have shown that caffeine abolishes the delay, but this is not entirely due to inhibition of Rad3 activity, since a *rad3 *mutation does not abolish all of the G1/S delay. Since we have not identified a checkpoint mutation which abolishes the delay, we attribute the effect of caffeine to another effect than the inhibition of checkpoint proteins. Interestingly, recent data suggest that caffeine inhibits checkpoint responses without inhibiting the ATR and ATM kinases in human cells [51,52].

Previously, Rhind and Russell [53] showed that UV-irradiation during G1 delays passage through S-phase. However, this checkpoint arrests cells in S phase, requires Cds1 function and probably represents the intra-S checkpoint.

We have recently discovered a mechanism that delays entry into S phase in cells irradiated in early G1 in synchronised or in cycling cells [41] This inhibitory mechanism has several features in common with that described here. Both pathways are activated in early but not in late G1; both inhibit entry into S phase; both pathways are independent of classical checkpoint genes and of Cdc2 phosphorylation. These similarities argue that the G1/S mechanism demonstrated in germinating spores and in cycling cells is one and the same.

In budding yeast there is a G1 DNA damage checkpoint response that depends upon Mec1 [31,32,54], a homologue of the mammalian ATM/ATR and the fission yeast Rad3 protein. However, the budding yeast G1 checkpoint response depends on Rad53 [55,56], whereas its homologue in *S. pombe*, Cds1, is not involved in the present pathway. The budding yeast G1/S checkpoint delays entry into S phase by phosphorylating and thereby downregulating Swi6, the homologue of Cdc10 [57]. In contrast, in fission yeast Cdc10 dependent transcription is not delayed during the G1/S delay (Fig. 8).

Other possible mechanisms for the G1/S delay include inhibition of Cdc2 by Rum1 or an as yet unidentified mechanism such as preventing the formation of Cdc2-cyclin complexes or by restricting the availability of cyclins. We have shown that Rum1 is expressed during the delay and is required for the G1 delay. This observation does not imply that Rum1 is a direct target of the G1/S delay, but this remains an attractive possibility. Another possible mechanism for the delay is delaying expression of proteins required for the initiation of DNA replication. In particular, the findings that (1) irradiation in late G1 does not cause a delayed entry into S phase, (2) increased transcription of Cdc10 dependent genes in *res2Δ *overrides the delay, (3) transcription of Cdc10 dependent genes is not downregulated during the delay and (4) expression of Cdt1 is delayed, suggest that the G1/S delay is caused by delayed expression of Cdt1 and probably also of Cdc18 and Cig2. Since we have shown that transcription of Cdc10 regulated genes is not downregulated, the most likely mechanism of the delay is reduced translation rate of Cdt1 and possibly other proteins required for initiation of DNA replication.

## Conclusions

We studied the response of *Schizosaccharomyces pombe *cells to UV irradiation in G1. We used germinating spores to exploit a natural phenomenon where the cells have a long G1. In this paper we provide evidence for the existence of a mechanism in fission yeast that delays entry into S phase upon UV irradiaton in early G1. The G1 delay is independent of classical checkpoint proteins and Cdc2 phosphorylation. Our results point to a mechanism that delays translation of proteins required for S phase entry.

## Methods

### Fission yeast strains and methods

All our strains are derivatives of the *Schizosaccharomyces pombe *L972h^- ^strain. All basic growth and media conditions were as described [58].

### Sporulation and spore germination

Diploids were made by interrupted mating of *h*^+ ^and *h*^- ^strains carrying the *met3 *or *ade1 *complementing markers. The *rum1:::ura4*^+^/*rum1*^+ ^diploid was made by protoplast fusion since *rum1Δ *is sterile [44]. All diploids, with the exceptions of *res2Δ *(which is deficient in meiosis [59]) and *rum1Δ*, were homozygous for the respective mutations. In case of these two mutants *Δ/wild type ura4-D18*/*ura4-D18 *diploids were sporulated and the spores were germinated in the absence of uracil. The diploids were sporulated in liquid malt extract medium at 30°C, then incubated with 3 μl/ml β-glucuronidase (*Helix pomatia *juice, Biosepra) at 30°C overnight. The spores were washed twice in water and resuspended in EMM2 supplemented with adenine and methionine for germination and UV irradiation.

### UV irradiation

Cells were irradiated with 254 nm UV light while rapidly stirred in a thin layer (3 mm) of liquid medium. The dose administered was measured with a radiometer (UVP instruments) and an exposure time of 4 minutes gave an incident dose of about 1100 J/m^2^. Cell survival was monitored by conventional plating on YE plates. The incident dose does not reflect the dose absorbed by the cells because UV light of this wavelength penetrates poorly into water. However, since irradiation conditions were constant, the incident dose was proportional to the absorbed dose.

### Protein extracts and western blots

Protein extracts for western blotting were made by TCA extraction, as described previously [19]. For western blot analysis the following antibodies were used: anti-phosphotyrosine Cdc2 (Sigma C0228) at a dilution of 1:400, anti-PSTAIRE against Cdc2 (Santa Cruz sc-53) at a dilution of 1:2000, anti-myc (PharMingen) at a dilution of 1:1000. The secondary antibodies were either HRP or AP conjugates, used at a dilution of 1/5000. Detection was performed using the enhanced chemiluminescence procedure (NEN ECL kit). Cdc2 and phosphorylated Cdc2 was measured using ECF detection (Amersham) and quantified with the Image Quant software.

### RNA preparation and blotting

Total RNA was isolated as described [58], resolved on formaldehyde agarose gels and blotted onto a nitrocellulose membrane (NitroPure, Osmonics). All blots were hybridized with ^32^P-labelled RNA probes, generated with T7 RNA polymerase (Riboprobe System T7 Kit, Promega). For *cig2 *and *cdc18*, the ORFs were inserted into pGEM-3 MCS to serve as template for producing the RNA probes. For *cdt1*, a PCR fragment of the ORF with T7 promoter sequence attached to the lower primer was used as template. Hybridisation was carried out using standard procedures and visualised by a STORM 860 Phosphoimager (Molecular Dynamics).

### Flow cytometry

About 10^7 ^cells were spun down for each sample and fixed in 70% ethanol before storing at 4°C. Samples were processed for flow cytometry as described [60] and stained with Sytox Green (Molecular Probes S-7020) [61], and analysed with a Becton-Dickinson FACSCalibur. The fraction of 1C cells was quantified using the CellQuest software (BD Biosciences).

## Authors' contributions

EAN showed the existence of the delay and investigated the roles of Rad3, Chk1 and Cds1, Cdc2 phosphorylation and Cdc10-dependent transcription as a potential mechanism. MS, TT and HV investigated the roles of further checkpoint proteins and that of Res2. EB participated in the design and coordination of the study and in writing the manuscript. BG devised the study, analysed Rum1 and Cdt1 expression and drafted the manuscript. All authors read and approved the final manuscript.
